# Seed storage proteins of the globulin family are cleaved post-translationally in wheat embryos

**DOI:** 10.1186/1756-0500-5-385

**Published:** 2012-07-28

**Authors:** Adam G Koziol, Evelin Loit, Melissa McNulty, Amanda J MacFarlane, Fraser W Scott, Illimar Altosaar

**Affiliations:** 1 Department of Biochemistry, Microbiology and Immunology, Faculty of Medicine, University of Ottawa, Ottawa, K1H 8M5, Canada; 2 Present address: Department of Field Crops and Grassland Husbandry, Estonian University of Life Sciences, Kreutzwaldi 5, Tartu, 51014, Estonia; 3 Present address: Department of Biochemistry and Molecular Biology, Mayo Clinic, Rochester, MN, 55905, USA; 4 Ottawa Hospital Research Institute, Ottawa, Ontario, K1H 8L6, Canada; 5 Present address: Nutrition Research Division, Food Directorate, Health Canada, Ottawa, Ontario, K1A 0K9, Canada

**Keywords:** Globulin 3, Type 1 diabetes, Vicilin, Wheat, Allergies, Post-translational processing, Mass spectrometry, Seed storage protein, Celiac disease

## Abstract

**Background:**

The 7S globulins are plant seed storage proteins that have been associated with the development of a number of human diseases, including peanut allergy. Immune reactivity to the wheat seed storage protein globulin-3 (Glo-3) has been associated with the development of the autoimmune disease type 1 diabetes in diabetes-prone rats and mice, as well as in a subset of human patients.

**Findings:**

The present study characterized native wheat Glo-3 in salt-soluble wheat seed protein extracts. Glo-3-like peptides were observed primarily in the wheat embryo. Glo-3-like proteins varied significantly in their molecular masses and isoelectric points, as determined by two dimensional electrophoresis and immunoblotting with anti-Glo-3A antibodies. Five major polypeptide spots were identified by mass spectrometry and N-terminal sequencing as belonging to the Glo-3 family.

**Conclusions:**

These results in combination with our previous findings have allowed for the development of a hypothetical model of the post-translational events contributing to the wheat 7S globulin profile in mature wheat kernels.

## Background

The 7S globulins, orthologs of the vicilins of the Leguminoseae, are salt-soluble storage proteins that accumulate during seed development [1,2]. Vicilins were first described by Osborn and Campbell in 1898 as a class of seed storage proteins in *Vicia faba* (horse bean) [3]. Both the vicilins and the legumins, distinguishable by their sedimentation coefficients of 7–9S and 11–13S, respectively [4], contain characteristic β-barrel cupin domains [5]. The 7S globulins are translated as preproproteins that, following the co-translational cleavage of the signal peptide, assemble into homo or heterotrimers [6] within the lumen of the endoplasmic reticulum [7]. Prior to storage in seed protein storage vesicles, the trimers undergo post-translational processing, which includes glycosylation and partial endoproteolytic cleavage [8,9].

Exposure to certain wheat seed proteins can induce a number of immune-mediated diseases including gluten sensitive enteropathy (celiac disease) [10], Baker’s asthma and wheat-dependent exercise-induced anaphylaxis (WDEIA) in predisposed individuals [11]. The *Triticum aestivum* (wheat) storage protein WP5212, later named globulin-3A (Glo-3A), has been demonstrated to be a potential food allergen [12], identified as the first candidate wheat protein associated with the development of type 1 diabetes (T1D) [13], and now celiac disease [14]. We recently identified the genomic origins of three *Glo-3* genes, *Glo-3A, B* and *C* in the wheat cultivar Glenlea [15]. Immunofluorescence studies have localized the Glo-3 gene products to the developing wheat seed embryo and aleurone layer [15].

Few studies have sought to characterize wheat 7S globulins because they were thought to be minor storage proteins with little contribution to the bread-making properties of wheat flour [16,17]. However, 7S proteins, based on their sedimentation coefficient, have been characterized in barley and maize, and more recently, two *Glo-3*-like sequences have been identified in the model cereal *Brachypodium distachyon*[18]. In addition to cultivar Glenlea, Glo-3 proteins have been observed in cultivars Butte 86 and Recital [19,20], indicating that Glo-3 is well-conserved in wheat, thus deserving more attention.

Due to its potential association with T1D, we initiated the present study to characterize the Glo-3-related proteins and peptides in wheat cultivar AC Barrie, the original source of WP5212 [13]. We hypothesized that Glo-3 undergoes post-translational processing, including glycosylation and endoproteolytic processing, similar to 7S proteins in other species. Therefore, we sought to characterize the expression and the distribution of Glo-3 antigenically related proteins by M_r_ and pI in the embryo and endosperm of AC Barrie, and to link observed protein fragments with their corresponding endoproteolytic cleavage events.

## Results

### Glo-3 antigenically-related proteins co-isolate with the wheat globulin fraction

**Figure 1:**
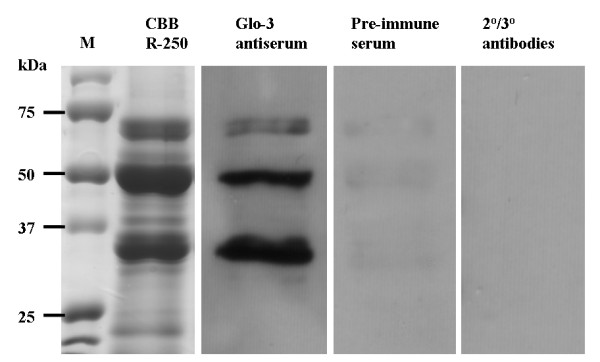
**SDS-PAGE and immunoblot analysis of AC Barrie salt-soluble proteins.** The salt-soluble fraction from AC Barrie seeds was separated under reducing conditions by SDS-PAGE (12% polyacrylamide) and stained with CBB R-250. Standard lane (M) is Precision Plus Protein (Bio-Rad). Proteins were immunoblotted with polyclonal anti-Glo-3A antiserum at a 1:10,000 dilution, with pre-immune serum (1:10,000), or with secondary and tertiary antibodies alone.

To characterize the Glo-3 antigenically-related proteins in whole AC Barrie seeds, globulins were extracted, following the classical method [21,22]. The globulin-enriched fraction was separated by 1D SDS-PAGE and immunoblots were probed with polyclonal rabbit antibodies specific for Glo-3A (Figure 1) [15]. The four most intense protein bands, as resolved by SDS-PAGE, had relative mobilities of 33–36, 47–53 and 64–65 and 66–68 (doublet) kDa. The Glo-3 antigenically-related proteins had comparable M_r_ to these intense bands (33–37, 47–53, 64–68 kDa). Pre-immune serum and secondary/tertiary antibody controls were negative for immunoreactivity with the Glo-3-related proteins (Figure 1).

### The Glo-3-related proteins are primarily located in the embryo

**Figure 2:**
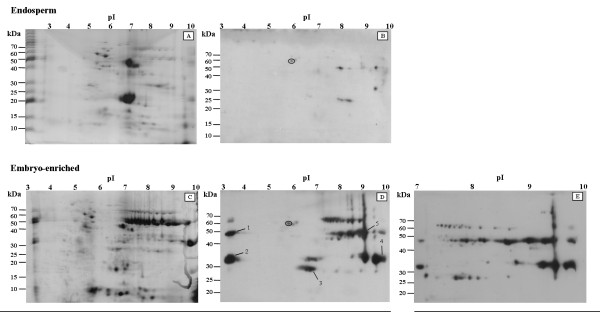
**Globulin diversity is greater in the embryo-enriched fraction than in the endosperm as observed by 2-dimensional electrophoresis.** Salt-soluble globulins were extracted from AC Barrie wheat seed endosperm (panels **a**, **b**) and embryo-enriched (panels **c**, **d**, **e**) fractions and separated by 2-DE. Proteins were stained with CBB R-250 (panels **a**, **c**) or transferred to nitrocellulose and probed with polyclonal rabbit anti-Glo-3A antiserum (panels **b**, **d**, **e**). Marker lanes (M) are Pre-stained Benchmark (Invitrogen). Molecular masses shown on immunoblots are approximations. Spots chosen for mass spectrometry are labeled 1–5 and marked with arrows, and represent a sampling of the major observed molecular masses ( ~30 kDa and ~50 kDa) with isoelectric points in the acidic (pH 3), neutral (pH 6–7) and basic (pH 9–10) regions. Circled spots are non-specific spots common to blots probed with pre-immune serum and anti-Glo-3A antibodies.

Protein expression levels of 7S globulins have been shown to be highest in the embryo and aleurone layers, while almost absent in the endosperm [23-25]. To study the expression of Glo-3 proteins, AC Barrie endosperm and embryo salt-soluble protein fractions were compared by two-dimensional (2D) electrophoresis according to pI and M_r_, followed by immunoblotting using anti Glo-3A antibodies (Figure 2). The embryo protein fraction was noticeably more complex than the endosperm fraction, with 287 spots detected by GE Healthcare ImageQuant TL Colony Version 7.0 in the CBB R-250-stained 2D polyacrylamide gel of the embryo protein fraction compared to the 122 spots detected in the 2D gel of the endosperm protein fraction (Figure 2, panels A, C). Analysis of the immunoblots revealed 91 spots corresponding to antigenically-related Glo-3-related proteins in the embryo protein fraction, and 46 spots in the endosperm protein fraction (Figure 2, panels B, D). On the basis of the increased anti-Glo-3 immunoreactivity with the salt-soluble embryo protein fraction, further studies focused on the AC Barrie embryo. One immunoreactive spot, with M_r_ 57 kDa and pI 5.8, was common between blots probed with anti-Glo-3A specific antibodies (circled in Figure 2, panels B and D) and with pre-immune serum (data not shown). This spot was considered non-specific for Glo-3 immunoreactivity.

Of the 91 anti-Glo-3 immunoreactive spots in the salt-soluble embryo protein fraction, 59 spots corresponded to the four dominant bands identified in the 1D immunoblot (M_r_ 33–37, 47–53, 64–65, and 66–68 kDa) (Figure 1 and Figure 2, panel D). Twelve spots in the M_r_ 33–37 kDa range and 23 spots in the M_r_ 47–53 kDa range were observed with 20 spots having pI values between 7.5 and 9.5. Twenty-four spots were in the M_r_ range of the 64–65 and 66–68 kDa doublet, with 21 spots having pI values between 7.5 and 9.5. As 71 of the 91 spots had pI values between 7 and 10, the salt-soluble embryo protein faction was resolved on a 2D gel with a pH range of 7–10 (Figure 2, panel E). There were 103 Glo-3 immunoreactive spots identified within this narrower pH range, with 70 of the 103 spots with M_r_ of 33–37, 47–53, 64–65, or 66–68 kDa.

### Identification of selected Glo-3-related polypeptides

To confirm that the antigenic epitopes detected by the anti-Glo-3A antibodies were specific to Glo-3, five anti-Glo-3-immunoreactive spots from the salt-soluble embryo protein fraction were excised from the 2D gel, and analyzed by mass spectrometry (LC-MS/MS) (Figure 2, panel D; numbers indicate location of spots). The spots excised were chosen as they had a wide range of M_r_ and pI values, and they fell outside the predicted M_r_ and pI values of proglobulin-3 (GenBank Accession JQ945759) (M_r_ 66.6 kDa and pI 8.5, as calculated using the Expasy Compute pI/Mw tool). Mass spectrometry results are summarized in Table 1. All five spots were identified as Glo-3A by interrogating the non-redundant NCBI database.

**Table 1:** MS/MS sequencing results of selected gel spots of salt-soluble 7S wheat globulins

**Spot ID**	**Protein name (GenBank accession)**	**Mascot score**	**% Coverage**	**Peptides**
1	globulin-3A (JQ945759)	210	17.5	VFGPRSF; DEVSRLL; HTISVPGKF; GRPAREVQEVF; RVAIMEVNPRAF; TVRQGDVIVAPAGSIMHL; VVPPGHPVVEIASSRGSSNL; VAQGEGVLTVIENGEKRSY
2	globulin-3A (JQ945759)	131	12.9	VFGPRSF; DEVSRLL; VVPGLTDADGVGY; RVAIMEVNPRAF; TVRQGDVIVAPAGSIMHL; VAQGEGVLTVIENGEKRSY
3	globulin-3A (JQ945759	38	3.4	VVPPGHPVVEIASSRGSSNL
4	globulin-3A (JQ945759)	135	13.8	RPFDEVSRLL; RVAIMEVNPRAF; VAQGEGVLTVIENGEKRSY; SAKPLLASL; TVRQGDVIVAPAGSIMHL; VVPGLTDADGVGY
5	globulin-3A (JQ945759)	144	17.7	DEVSRLL; RPFDEVSRL;VVPGLTDADGVGY; RVAIMEVNPRAF; EINAERNERVWL; TVRQGDVIVAPAGSIMHL; VVPPGHPVVEIASSRGSSNL; VAQGEGVLTVIENGEKRSY

### Characterization of selected Glo-3-related polypeptides

To study the post-translational processing of Glo-3, three spots were analyzed with N-terminal sequencing. One sequence (Spot 1) may be N-terminally blocked because no information could be obtained, despite protein visualization after amido black staining. The N-terminal sequence of Spot 3 was determined to be SRDTFNLL, which matched the Glo-3A (GenBank Accession JQ945759) sequence starting at amino acid residue 337. Spot 4 was difficult to visualize following protein transfer and staining. As determined by N-terminal sequencing, the first two residues could not be resolved (X) and the last residue was reported as either arginine (R) or glutamic acid (E). Using arginine as the last residue, the resulting sequence XXHGDSRR matched the findings of Singh et al. [26], and the Glo-3B sequence (GenBank accession FJ439136) starting at residue 117.

**Figure 3:**
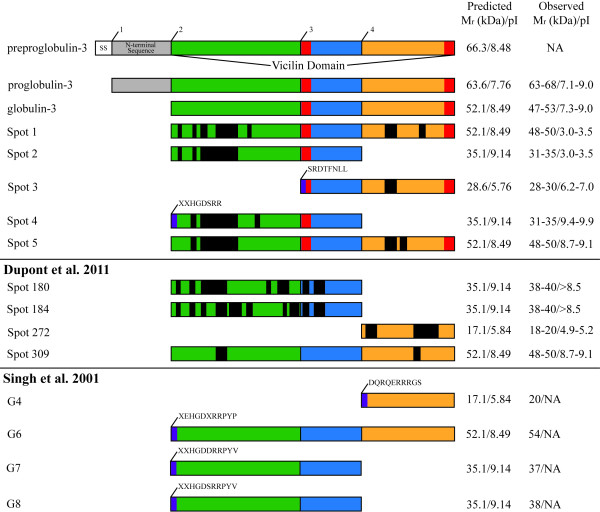
**Model of Glo-3 endoproteolytic processing.** The observed M_r_, pI, MS/MS sequence data, and N-terminal sequence data from this study and previous studies [26,58] were reconciled with theoretical peptides created by the endoproteolytic cleavage of preproglobulin-3 (GenBank Accession JQ954759). A linear representation of preproglobulin-3 is shown with approximate locations of potential cleavage sites, labeled 1–4. The protein domains are represented as follows: signal sequence (SS) (white); N-terminal sequence (grey); vicilin domain (divided into three segments by cleavage sites – N-terminal segment (green), middle segment (blue), C-terminal segment (orange). Red boxes correspond to the location of the linear epitopes used when creating the anti-Glo-3A polyclonal antibodies. Black boxes correspond to the location of MS/MS sequenced peptides. Purple boxes correspond to sequence obtained from N-terminal sequencing. The observed and expected molecular masses (kDa) and isoelectric points (pI) of the resulting polypeptides following endoproteolytic cleavage is indicated on the right. Additionally, the expected processing of proteins inferred by previous studies (Dupont et al., 2011 [20] and Singh et al., 2001 [26] have been demonstrated.

The post-translational endoproteolytic cleavage events of preproglobulin-3 that would be required to yield polypeptides with M_r_ and pI corresponding to the sequenced spots are summarized in Figure 3. In addition to the M_r_ and pI of the spots in Figure 2, panel D, the location of the MS sequenced peptides within globulin-3 (black bars), N-terminal sequence data (purple bars - when available), as well as the location of the epitopes used to generate the polyclonal anti-Glo-3A antibodies (red bars) were considered. The size and location of the signal sequence was predicted by TargetP 1.1 [27,28], (http://www.cbs.dtu.dk/services/TargetP/). Proteins from previous studies (Dupont et al., 2011 [20] and Singh et al., 2001 [26] were included to demonstrate that the methods used for the determination of the processing events in the current study are applicable to previously published findings and that our observed processing corresponds to the processing inferred in the literature. The processing of Spot 3 (Figure 3) is supported by the N-terminal sequence that matched cleavage site 3 (SRDTFNLL), as well as the MS sequenced peptide in the C-terminal vicilin domain segment, and an observed M_r_ 28–30 kDa and pI 6.2-7.0. Spot 3 corresponds to the cleavage of proglobulin-3A at cleavage site 3, with predicted M_r_ 28.6 kDa and pI 5.76. The processing of Spot 4 is supported by N-terminal sequence data that matches the previously documented cleavage site 2 (XXHGDSRR) [26]. Spot 4 corresponds to cleavage of proglobulin-3A at cleavage sites 2 and 4, with predicted M_r_ 35.1 kDa and pI 9.14. Spot 2 lacked N-terminal sequence data, but as with Spot 4, contained MS sequenced peptides present in the N-terminal vicilin domain segment and an observed M_r_ 31–35 kDa. Cleavage at both sites 1 and 3, or 2 and 4 yield products with M_r_ of approximately 35 kDa. However, the epitopes used for the generation of the anti‐globulin-3A antibody are present in the middle and C-terminal vicilin segments. Neither of those epitope sites are present in products created from cleavage sites 1 and 3, so therefore Spot 2 corresponds to cleavage at sites 2 and 4. Spots 1, and 5 lack N-terminal sequence data, but had MS sequenced peptides that localized to the N-terminal and C-terminal segments of the vicilin domain, and exhibited relative mobilities of 48–50 kDa (Figure 3) as well. The pI of Spot 1 was 3.0-3.5, while the pI of Spot 5 was 8.7-9.1 Both Spot 1 and Spot 5 correspond to the theoretical cleavage of preproglobulin-3A at cleavage site 2 with predicted M_r_ 52.1 kDa and pI 8.49.

## Discussion

### Type 1 diabetes

Inflammation associated with the T-cell-mediated autoimmune disease T1D results in the loss of the insulin-producing β cells in the pancreatic islets of Langerhans [29]. While the incidence of T1D has steadily increased in developed countries over the past 60 years, a definitive cause of T1D has yet to be elucidated [30]. While many risk genes for T1D have been identified, it has been proposed that most T1D-related genes are not highly penetrant and that T1D is actually a complex disease requiring both genetic susceptibility and the exposure to environmental risk factors [31]. Serum IgG antibodies pooled from human patients with T1D, but not from matched control groups, were able to bind Glo-3A *in vitro*, indicating that Glo-3A may be immunogenic in certain individuals with T1D [13]. We therefore chose to characterize, in more detail, the Glo-3-related proteins in the wheat cultivar AC Barrie with respect to protein maturation in developed seeds.

### Characterization of Glo-3

When the amino acid sequences of preproglobulin-3A from the wheat cultivars AC Barrie and Glenlea are compared, there are five amino acid substitutions out of 588 residues [15]. However, the theoretical isoelectric points of these Glo-3A proteins are 8.48, and 7.78 in AC Barrie and Glenlea, respectively. The difference in pI of 0.70 is due to the substitution of two arginine residues (R43Q and R102H) in the AC Barrie Glo-3A. The AC Barrie salt-soluble Glo-3-related proteins demonstrate a range of isoelectric points concentrated in the basic range (pH 7–9) when separated by 2DE (Figure 2, panel E). As there are three *Glo-3* genes in the wheat cultivar Glenlea [15], it is feasible that AC Barrie would also have three *Glo-3* genes, as both AC Barrie and Glenlea are closely related hexaploid wheat cultivars [32]. The charge trains of gel spots with similar M_r_ and a range of pI values at the major size groups (Figure 2) are likely due to post-translational processing and modifications, as previously discussed [17], as well as amino acid substitutions between the multiple immunologically related Glo-3 proteins in AC Barrie, or from artifacts generated during the execution of the extraction and examination protocols [33].

When the wheat cultivar AC Barrie salt-soluble globulin fraction was resolved by 1D SDS-PAGE under reducing conditions, bands of three major size ranges of approximately 64–70 kDa, 47–53 kDa and 33–37 kDa were visible (Figure 1); referred to as 65 kDa, 50 kDa and 35 kDa, respectively. These three major wheat globulin groups are characteristic of the 7S globulins described in other studies [17,25]. Intriguingly, while Spot 3 had an observed M_r_ of 28–30 kDa (Figure 2D), no distinct band is visible in this region in Figure 1, though a faint band is visible just below the 35 kDa band. The 28–30 kDa band is likely less intense than the major bands, as Spot 3 is the only significant immunoreactive spot at 28–30 kDa, while the doublet at 65 kDa comprises several spots and is still markedly less intense than the 50 and 35 kDa bands, which both include several intense spots.

The 7S globulins are enriched in the embryo and aleurone layer of wheat and other cereals [9,21,34,35]. Consistent with our previous findings [15], immunoblots probed with anti-Glo-3A polyclonal serum revealed that 7S globulins are expressed at low levels in the salt-soluble fraction of wheat endosperm (Figure 1). In the embryo-enriched fraction, Glo-3 related proteins are restricted to each of the major size groups, suggesting that these Glo-3-related proteins have similar post-translational processing patterns as the previously characterized 7S globulins.

### Post-translational processing of Glo-3

The 7S storage proteins in other plants undergo a series of post-translational modifications which include limited endoproteolytic events [36-38]. Glycosylation is frequently observed in 7S globulins as N-linked complex glycans [39], but is not required for proper folding or export to the protein storage vacuole (PSV) [40]. A previous study has shown that wheat 7S globulins bind the lectin concanavalin A, although the exact nature and extent of this binding is unknown [17]. Additionally, other cleavage events may occur at the N-terminus as observed for maize Glb1 [41]. These post-translational modifications, among others, likely contribute to the heterogeneity of the observed isoelectric points as well as the wide range of observed molecular masses among the Glo-3-related proteins.

The MS/MS sequencing of the numbered spots in Figure 2, panel D, all returned Glo-3A sequences when queried against the non-redundant NCBI protein database, indicating that, similar to orthologous 7S storage proteins in other species [9], the peptide spots in Figure 2, panel D were derived from a common precursor protein. The Glo-3-related proteins found at the acidic end of the pH spectrum, including the proteins in spots 1 and 2, exhibited a pI of approximately 3.0 (Figure 2, panel D). The low pI of these proteins could be due to protein precipitation during the rehydration of the IPG strips [42], as similar spots are visible at pH 7.0 in the immunoblot of the 2D gel of pH 7–10 (Figure 2, panel E).

Considering features such as the location of the MS/MS sequenced peptides within Glo-3A, N-terminal sequence data (when available), as well as the M_r_ and pI of the spots, we were able to deduce the likely cleavage pattern of the preproglobulin-3 precursor protein (Figure 3) that resulted in the globulin-3 polypeptide spectrum recognized by anti-Glo-3A antibodies (Figure 2, panel D). It must be noted that the antibodies used to probe the globulin fraction were raised against two linear epitopes from the vicilin domain of WP5212: SRDTFNLLEQRPKIAN and RGDEAVEAFLRMATA [13,43]. These epitopes are located in the middle (blue) and C-terminal (orange) vicilin domain segments (Figure 3). Therefore, N-terminal polypeptides created by the endoproteolytic cleavage at site 3 may not be recognized by the anti-Glo-3A antibodies used in this study.

The model of endoproteolytic cleavage presented in Figure 3 suggests that the Glo-3-related proteins of varying molecular masses and isoelectric points originate from 7S globulin precursors possessing a signal peptide, an N-terminal segment and a vicilin domain (Figure 3). These precursors belong to a multigene family whose members differ slightly in sequence, with varying length N-terminal segments [4]. We propose that the Glo-3 protein, prior to co-translational removal of the signal sequence, is a ~66 kDa monomer (Figure 3). After signal sequence removal (cleavage site 1), the protein becomes a ~64 kDa holoprotein. Three internal cleavage sites have been identified that, when processed, can yield polypeptides with a range of M_r_ and pI values (Figure 3). Spot 3 (Figures 2, panel D, Figure 3) was observed at M_r_ 28–30 kDa and pI 6.2-7.0, and Spot 4, which has a similar M_r_ (31–35 kDa), a more basic pI (9.4-9.9), and a different N-terminus than Spot 3. The N-terminal sequence determined for Spot 3 SRDTFNLL represents a novel cleavage site for globulin-3. The location of this cleavage site is consistent with processing by vacuolar processing enzymes [44], as an aspartic acid residue precedes the N-terminal sequence of Spot 3. These findings suggest that these ~30 kDa globulin-3-related polypeptides could arise from different processing events of the same precursor, as was observed for barley Beg1 [9,35]. Curiously, there were no proteins identified in the immunoblotting experiments that corresponded to the product of processing at cleavage site 4, as was documented in other studies that sequenced globulin-3 (Figure 3 and [19,20,26]). Potentially, as the anti‐globulin-3A antibodies are polyclonal and were prepared by the co-immunization of rabbits with a mixture of two separate peptides [45], it is possible that the antibodies recognize only one of the two peptides. However, further study on the binding of these antibodies to processed globulin-3 proteins is still required in order to achieve a more definitive understanding.

When compared to the Glo-3A, Glo‐3B, and Glo-3C sequences previously published by our group [15], each of the spots contained at least one peptide in Table 1 that mapped solely to the Glo-3A coding sequence (GenBank Accession JQ945759 - data not shown). As the Glo-3A protein coding sequence is 99% identical between wheat cultivars Glenlea (GenBank accession ACJ65514) and AC Barrie (JQ945759), the Glo-3B and Glo-3C proteins may share a high percentage identity between these two cultivars. Therefore, we attributed all spots to Glo-3A. However, on close inspection of the Glo-3B (FJ439136) genomic sequence, there are several insertion/deletion events (data not shown) that would yield a protein of similar molecular weight as Glo-3A (66.3 kDa). Further sequencing of Glo-3 cDNA clones from both AC Barrie and Glenlea cultivars is necessary to determine the transcribed sequence of Glo-3.

The endoproteolytic processing events outlined here are likely part of a series of post-translational events that lead to the maturation of the Glo-3-like proteins, also similar to those observed for barley Beg1 [9]. The identification of wheat Glo-3A holoproteins (~65-70 kDa) by immunoblot analysis suggests that the endoproteolytic modifications described are partial and may not be prerequisites for proper folding, transport, targeting, and storage of the Glo-3 proteins. Alternatively, as the wheat genome has not been sequenced, the spots with M_r_ 65–70 kDa may correspond to proteins in the globulin-3 family that encode proteins recognizable by the antiglobulin-3A antibodies, but are processed in a different manner than the proteins observed in this study. Further sequencing and study are required in order to fully catalogue the globulin-3 family. The presence of polypeptides of ~50 kDa and ~30-38 kDa (in Singh et al. [26] and present study) with the same N-termini reinforce the idea that not every hypothesized set of endoproteolytic processing events occurs, and some could occur with varying degrees of processing. Such maturation of vicilins has not only medical implications, but also implications for the quality of foods rich in vicilins following processing and production (e.g. wheat, soybean [45,46]).

## Conclusion

With a greater understanding of the endoproteolytic processing events that lead to the maturation of the Glo-3 family of proteins observed in the salt-soluble embryo protein fraction, we can now refine the research of Glo-3 to certain domains present only in the polypeptides that are associated with T1D or celiac disease following endoproteolytic processing. In addition, the specific breeding or genetic modification of wheat could be performed to minimize any potential disease- or food quality-related protein or peptide content compared to that of existing wheat cultivars, as is underway for the deletion of the conglycinin α' subunit from the soybean proteome [47,48].

## Methods

### Wheat seed protein extraction and sample preparation

For each extraction, 4 g of whole *Triticum aestivum* AC Barrie seeds or 50–100 embryos or endosperm, which were dissected as in [49] were employed. Seeds were initially ground in a domestic coffee grinder. Finer powder was obtained by hand milling using a mortar and pestle under liquid nitrogen. Wheat powder was mixed with 10 volumes of fresh ice-cold acid denaturing solution (10% (w/v) trichloroacetic acid (TCA) and 0.05% (w/v) dithiothreitol (DTT) in acetone). Samples were stirred at 4 °C for 1 h and left at −20 °C overnight. The resulting suspension was centrifuged for 30 min at 35,000 x *g* at 4 °C (Beckman Avanti J-25, rotor JA 25.50). The supernatant was decanted and the pellet resuspended in ice-cold acetone containing 0.05% (w/v) DTT. The mixture was extracted by incubation for 1 h at −20 °C. The suspension was centrifuged for 20 min at 35,000 x *g* at 4 °C, the supernatant decanted and the pellet dried on ice. Dried powder was extracted with 15 ml of 1 M NaCl solution (1.0 M NaCl, 0.05 M Tris, pH 8.0). The mixture was stirred for 1 h at room temperature and centrifuged (27,000 x *g* for 30 min at 22 °C). Supernatants were collected and the extraction with 1 M NaCl solution was repeated. The supernatants were pooled and dialysed against 5 changes of ddH_2_O for 24 h at 4 °C. Precipitates were collected by centrifugation at 35,000 x *g* at 4 °C for 45 min. Pellets were drained and extracted for either one-dimensional (1D) or two-dimensional protein separation.

### Sample preparation for 1D separation

Pellets were resuspended in 1 M NaCl solution. Samples were centrifuged at 20,000 x *g* for 1 h at 22 °C and quantified by the bicinchoninic acid (BCA) assay [50]. Supernatants were aliquoted and stored at −80 °C until use.

### Sample preparation for 2D separation

Proteins from pellets were extracted with 1.5 – 3 ml rehydration buffer (8 M/2 M deionized urea/thiourea, 2% 3-([3-cholamidopropyl]dimethylamino)-1-propanesulfonate (CHAPS), 50 mM DTT, 0.0005% bromophenol blue) containing pH 3–10 ampholytes (Bio-Rad). Following sonication, samples were centrifuged at 200,000 x *g* for 1 h at 22 °C in the Beckman TL-100 ultracentrifuge. Supernatants were collected, quantified by Bradford method [51], aliquoted and stored at −80 °C until use. Throughout the 2D separation process, care was taken not to heat the urea/thiourea-containing solutions above 30 °C to avoid carbamylation of amino groups which can lead to artifactual spot heterogeneity [52].

### 1D SDS-PAGE protein fractionation

Protein extracts were fractionated under SDS-PAGE reducing conditions. Protein samples were combined in a 1:1 (v/v) ratio with 2X sample buffer (4% SDS, 20% glycerol, 0.12 M Tris (pH 6.8), 10% (v/v) β-mercaptoethanol, 0.01% bromophenol blue). Samples were boiled for 5–10 min and centrifuged at 20,000 x *g* for 10 min. All samples were loaded on discontinuous (5% stacking, 10%-12% resolving) 1.5 mm SDS polyacrylamide gels [53]. The SDS running buffer, adjusted to pH 8.3 with NaOH, consisted of 25 mM Tris base, 0.19 M glycine and 0.1% SDS. Electrophoresis was performed in the Mini-PROTEAN 3 System (Bio-Rad) at 100–150 V (400 mA) until dye front reached the bottom of the gel. Gels were either stained with Coomassie Brilliant Blue (CBB) R-250 or were used for immunoblot analysis.

### Immunoblot analysis

Proteins from SDS-PAGE were transferred under semi-dry conditions by means of the Trans-Blot SD Semi-Dry Transfer Cell (Bio-Rad). Gels were rinsed 5–10 min in semi-dry transfer buffer (24 mM Tris, 192 mM glycine and 15% methanol) and transferred to nitrocellulose membranes (Bio-Rad) also soaked in transfer buffer. Electroblotting proceeded for 1–1.5 h at 11 V (400 mA). Once complete, the transfer was verified by staining membranes with Ponceau S (0.2% (w/v) in 1% glacial acetic acid) followed by several washes of ddH_2_O. Prior to immunoblotting, membranes were destained with TBST buffer (10 mM Tris–HCl (pH 7.3), 0.1 M NaCl and 0.5% Tween-20) and were incubated 30 min with shaking, in 5% skim milk powder in TBST buffer at room temperature. Membranes were incubated with polyclonal rabbit anti-Glo-3A antibody [13] (diluted 1:10,000 in 5% skim milk powder in TBST and 0.05% NaN_3_) and left overnight at 4 °C with gentle rocking. Next day, membranes were washed 4 x 5 min in TBST, and incubated 1 h in biotin-SP-conjugated AffiniPure goat anti-rabbit IgG (H + L) secondary antibody (Jackson ImmunoResearch Laboratories, Inc., West Grove, PA) with a dilution of 1:100,000. This incubation was followed by another set of washes in TBST and a final incubation in affinity purified goat anti-biotin-horseradish peroxidase (HRP)-conjugated tertiary antibody (Cell Signaling Technology, Inc. Danvers, MA) for 1 h. Following 4 x 5 min washes, membranes were treated with ECL™ Western Blotting Detection Reagents (Amersham Biosciences, Piscataway, NJ) for 1 min, exposed to Kodak BioMax Light Film (Fisher Scientific, Nepean, ON). Film was developed by the Kodak X-OMAT 2000A Processor.

### Two-dimensional gel electrophoresis (2DE)

Proteomic analysis of the AC Barrie salt-soluble proteins was conducted by 2DE. The proteins were separated in the first dimension by isoelectric focusing (IEF) according to their isoelectric points (pI). Extracts were applied to linear immobilized pH gradient (IPG) 7 cm strips with pH ranges 3–10 and 7–10 (Bio-Rad, Mississauga, ON). A protein load of 100–150 μg was applied to strips for staining and immunoblot applications; 250 μg for mass spectrometry and N-terminal sequencing applications. Strips were covered in mineral oil and rehydrated overnight in a reswelling tray in 125–150 μl volumes of sample diluted in rehydration buffer. For strips in the pH ranges 3–10, DTT was used as a reducing agent; tributylphosphine (TBP) was used for strips in the pH range of 7–10. Rehydrated strips were focused in the PROTEAN IEF Cell (Bio-Rad) according to the following program: step 1, 250 V (30 min), linear ramp; step 2, 4000 V (2 h), linear ramp; step 3, 4000 V (10,000 Vh), rapid ramp. Immediately following isoelectric focusing, the strips were either stored at −80 °C for future use or equilibrated prior to running the second dimension. Strips were thawed, if necessary, and reduced for 15 min in 2 ml of equilibration buffer (6 M urea, 2% SDS, 20% glycerol, 0.375 M Tris–HCl, pH 8.8 containing 2% (wt/vol) DTT or 5 mM TBP). A second step, one of alkylation, was performed for 15 min in the same equilibration buffer with the exception that 2.5% (w/v) iodoacetamide was substituted for reducing agents DTT or TBP. Overlay agarose (Bio-Rad) was used to seal the equilibrated strips at the top of 1 mm vertical resolving gels (10% or 12% polyacrylamide). Second dimension SDS-PAGE was performed using the Bio-Rad MiniProtean 3 at 100 V (400 mA); runs were terminated when the bromophenol dye front had reached the end of the gel. Protein spots were counted using ImageQuant TL Colony Version 7.0 (GE Healthcare).

### Liquid chromatography tandem mass spectrometry (LC-MS/MS)

Gels were carefully manipulated under a laminar flow hood at all times to reduce keratin contamination. Following separation in the second dimension, proteins were detected with Bio-Safe Coomassie (Bio-Rad) according to manufacturer’s instructions and destained with multiple changes of ddH_2_O. Selected spots were excised from the gel by means of a clean scalpel blade and subjected to mass spectrometric analysis (Ottawa Institute of Systems Biology, OISB). Proteins were digested in-gel with chymotrypsin as previously described [54]. Trypsin digests were avoided because of the high frequency of arginine and lysine residues in the reported wheat globulin sequences [15,34]. Peptides were separated by liquid chromatography on an Agilent 1100 Series HPLC System (Agilent Technologies, Palo Alto, CA) and applied by electrospray to a QSTAR Pulsar quadrupole-TOF mass spectrometer (ABI/MDS Sciex, Concord, ON) as described in [55]. Resulting peptide masses were used to interrogate the non-repetitive NCBI protein database (06/10/2011; 1,4,324,397 sequences; 4,906,523,086 residues) using Mascot software (version 2.3) (Matrixscience Ltd.) as in our previous studies [56,57]. Fixed modifications were set for carbamidomethyl (C) and variable modifications for oxidation (M). One missed cleavage was allowed. Peptide and MS/MS mass tolerances permitted were ±100 ppm and 0.2 Da, respectively.

### N-terminal sequencing

Gels used for N-terminal sequencing were treated for wet transfer to PVDF membranes. Briefly, the gels were soaked in CAPS (3-(cyclohexylamino)-1-propanesulfonic acid) electroblotting buffer (10 mM CAPS/NaOH pH 11 and 10% methanol) for 5 min. Sequiblot PVDF (Bio-Rad) membranes were wet in 100% methanol, soaked in CAPS electroblotting buffer along with Whatman 3MM paper sheets and transblot sponges. The blotting sandwich was assembled and run in the Mini Trans-Blot Cell (Bio-Rad) at 50 V (170 mA) for 45 min. After disassembly, the membranes were rinsed thoroughly with ddH_2_O, saturated in 100% methanol and stained for a minimum of 1 min in amido black stain (0.1% amido black, 1% acetic acid and 40% methanol). Membranes were rinsed in multiple changes of ddH_2_O and air dried before excision of spots. Spots of interest were subjected to Edman degradation using the 494 cLC PROCISE Sequencing System (Applied Biosystems; Foster City, CA) for high sensitivity (femtomole quantities) N-terminal protein sequencing (University of Texas Medical Branch, UTMB, Biomolecular Resource Facility).

Abbreviations: (Glo-3), globulin-3; (WDEIA), wheat-dependent exercise-induced anaphylaxis; (T1D), type 1 diabetes; (TCA), trichloroacetic acid; (DTT), dithiothreitol; (PSV), protein storage vacuole; (1D), one-dimensional; (2D), two-dimensional; (BCA), bicinchoninic acid; (CHAPS), 3-([3-cholamidopropyl]dimethylamino)-1-propanesulfonate; (CBB), Coomassie Brilliant Blue; (HRP), horseradish peroxidase; (IEF), isoelectric focusing; (pI), isoelectric point; (IPG), immobilized pH gradient; (TBP), tributylphosphine; (OISB), Ottawa Institute of Systems Biology; (UTMB), University of Texas Medical Branch.

## Competing interests

The authors declare that they have no competing interests.

## Authors’ contributions

AGK performed bioinformatic analyses, analyzed data, assembled all figures and tables, drafted and edited the manuscript. EL helped draft the first manuscript, aided in editing, and helped with experiments. MM performed the experiments, contributed to the initial writing and subsequent editing. AJM, FWS provided the globulin-3A antibodies and edited the manuscript. FWS participated in the study design. IA designed the study, provided all reagents and edited the manuscript. All authors read and approved the final manuscript.
